# Supplementary material for: The histone chaperone CAF-1 prevents homologous recombination-mediated instability of the budding yeast ribosomal DNA during replication-coupled DNA double-strand break repair
Source: Nucleic Acids Res. 2025 Nov 27;53(22):gkaf1010. doi: 10.1093/nar/gkaf1010 (PMC12657057; doi:10.1093/nar/gkaf1010)
Supplement: gkaf1010_Supplemental_File [file gkaf1010_supplemental_file.pdf]

Figure S1

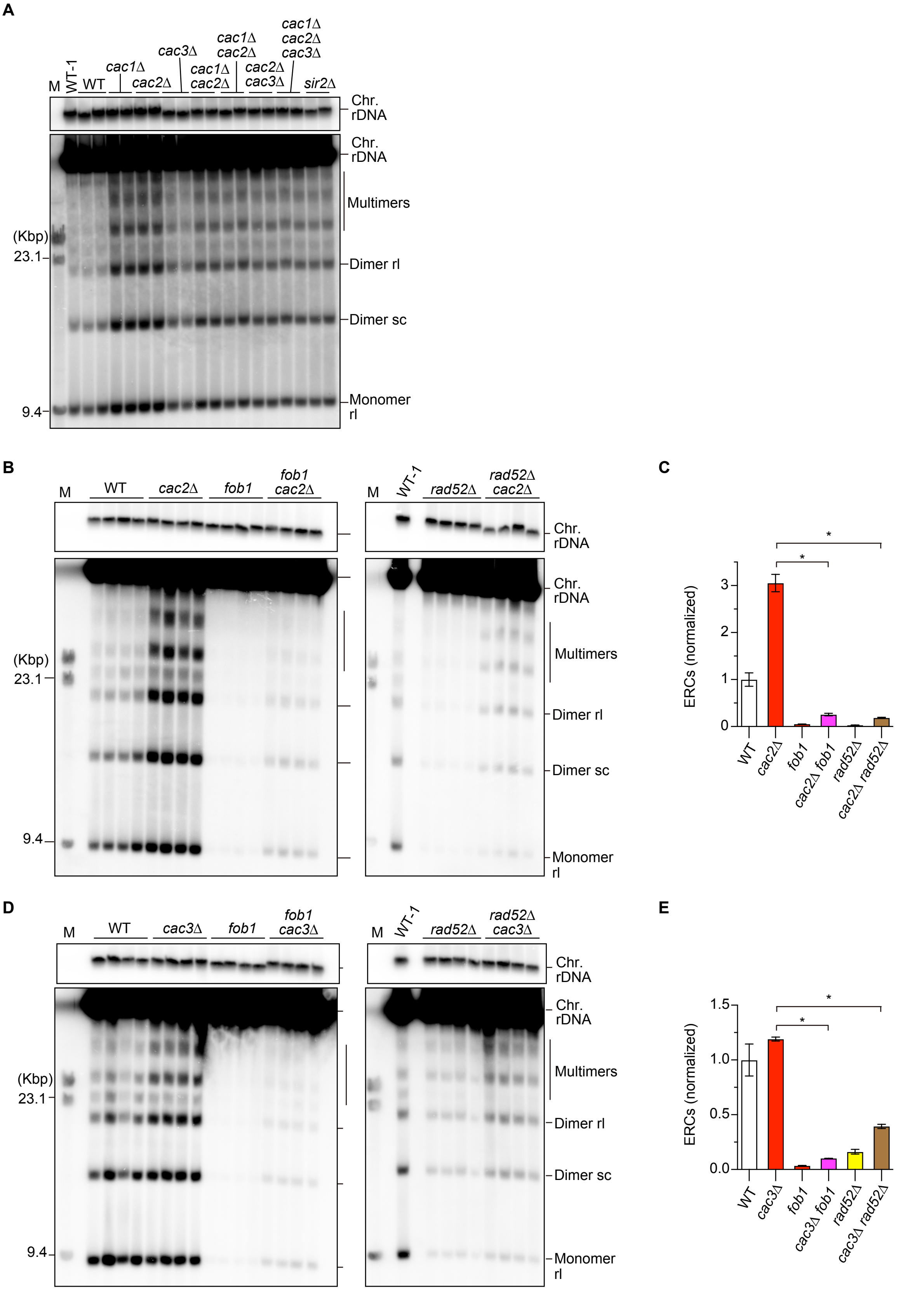

Supplementary Figure 1. ERC detection in the *caf-1* mutants.

(A, B, D) ERC detection. DNA was isolated from the two independent clones (A) and four independent clones (B, D) of the indicated strains and separated by agarose gel electrophoresis, followed by Southern blotting with the probe 1, as shown in Fig. 1A. Chromosomal rDNA and different forms of ERCs are indicated. rl and sc indicate relaxed and supercoiled ERCs, respectively. Supercoiled monomers ran off from the gel in the electrophoresis condition under these conditions. The sizes of lambda Hind III DNA markers are indicated. The top panel shows a short exposure of chromosomal rDNA signals. The DNA sample that was indicated as WT-1 was the same DNA sample isolated from the clone that was loaded a separate gel: WT-1 in (A) is the first WT clone in Fig. 1C; WT-1 in (B) is the first WT clone on the left gel; WT-1 in (D) is the first WT clone on the left gel. These DNA samples were used to normalize ERC levels in the mutants that were analyzed on separate gels. (C, E) Quantitation of ERCs. In (C) and (E), ERCs were quantified from (B) and (D), respectively. Multiple comparisons were performed by one-way ANOVA, followed by Tukey's multiple comparisons test; *, statistically significant difference ($p < 0.05$).

Figure S2

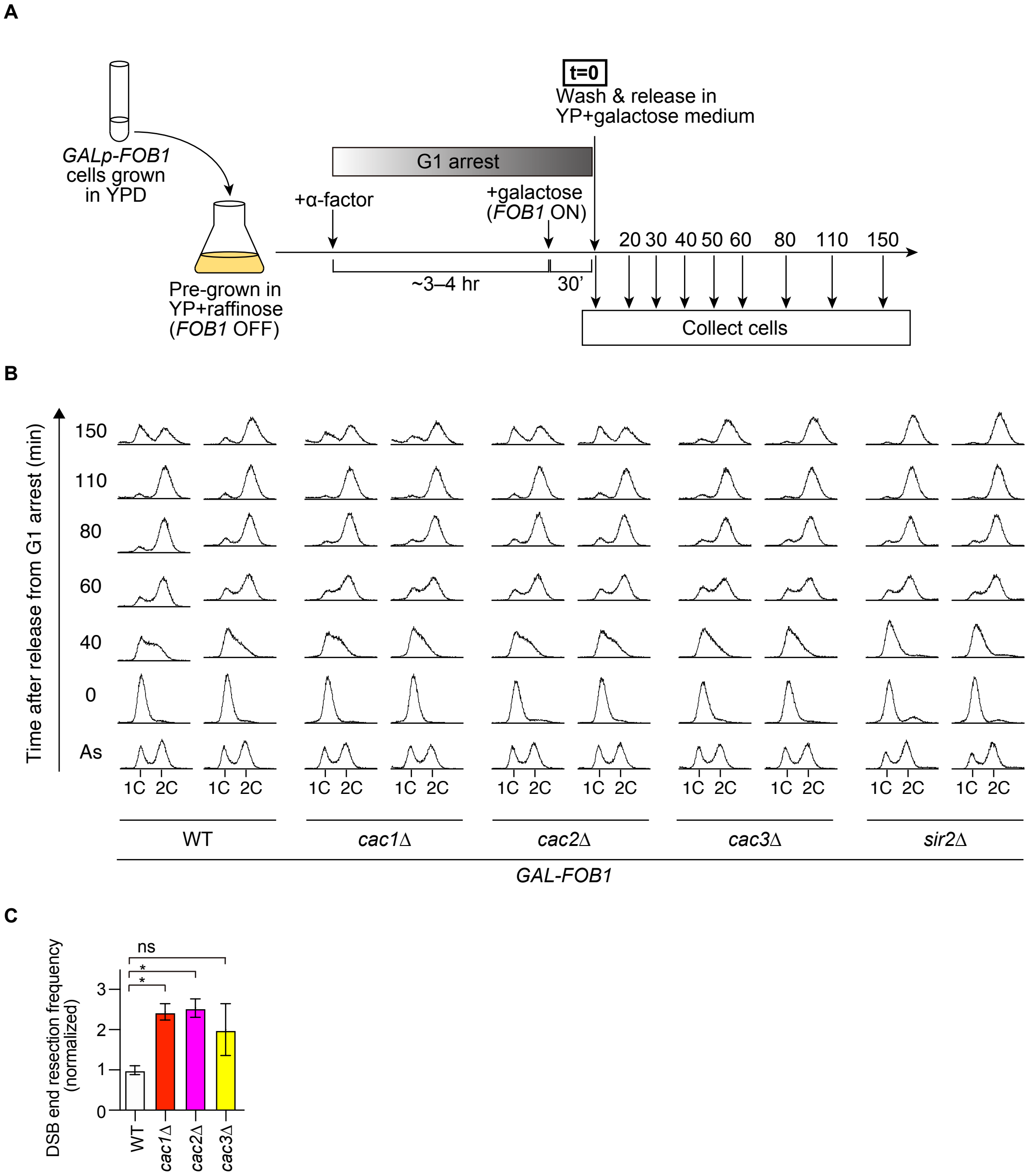

Supplementary Figure 2. Time course experiments to analyze formation and repair of DSBs.
(A) Outline of time course experiments.
(B) Cell cycle analysis of two representative time course experiments using GAL-FOB1 WT, *cac1*Δ, *cac2*Δ, *cac3*Δ, and *sir2*Δ strains by flow cytometry. Cells collected at different time points were stained with propidium iodide, and DNA content was analyzed. 1C indicates the DNA content of cells in G1 phase, representing one complete copy of the genome, while 2C corresponds to cells in G2/M phase, which have duplicated their DNA.
(C) Quantitation of the DSB end resection frequency in Fig. 3E. The average frequency of resected DSBs was determined and normalized to the average of WT clones (bars show the mean ± s.e.m.). DSB resection frequencies were compared between WT and caf-1 mutants by multiple comparisons using one-way ANOVA, followed by Tukey's multiple comparisons test; *, statistically significant difference ($p < 0.05$); ns, no significant difference.

Figure S3

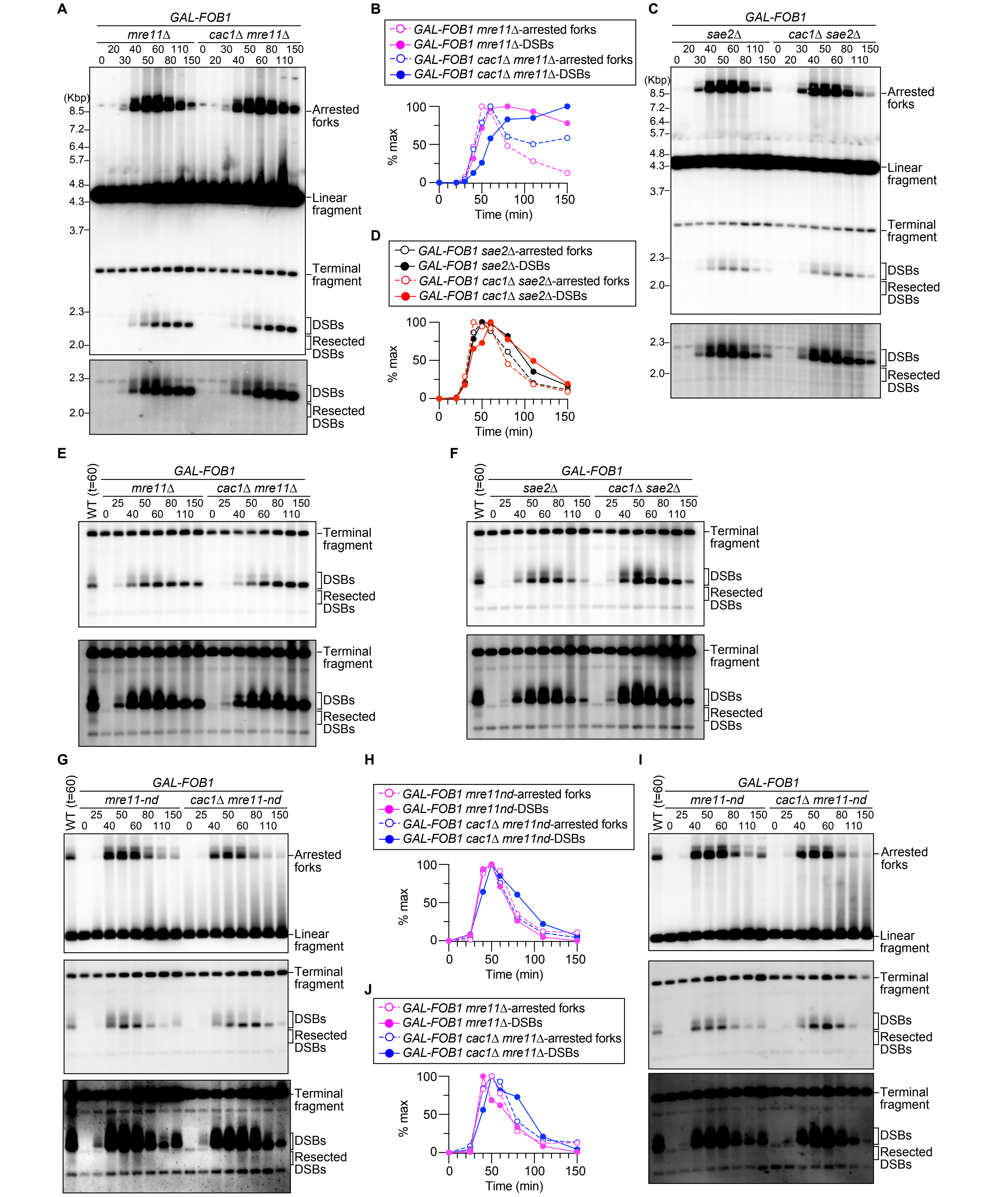

Supplementary Figure 3. Time course experiments to analyze DSB repair in the *cac1*Δ mutant deficient for the MRX complex functions.

(A, C, E, F, G, I) DSB assay. Time-course experiments were conducted, as described in Supplementary Figure S2A. Genomic DNA was isolated, digested with Bgl II, separated by size, and analyzed by Southern blotting with probe 4, as shown in Figure 3D. Arrested forks, linear fragments, DSBs and resected DSBs are indicated. Terminal fragments indicate the telomere-proximal rDNA repeat. Long exposure panels around DSBs and resected DSBs are shown below. In (E)–(H), DNA sample that was collected at the time point where resected DSB signal reached maximum in WT was loaded.

(B, D, H, J) In (B), (D), (H), and (J), arrested forks and DSBs are quantitated in (A), (C), (G), and (I), respectively, and expressed as percent of maximum values.

Supplementary Table 1. ***S. cerevisiae*** **strains used in this study**

Name	Genotype
HFY3	*MATa/α, cac1Δ::kanMX/CAC1, fob1::LEU2/FOB1, rad52Δ::hphMX/RAD52*
HFY4	*MATa/α, cac2Δ::kanMX/CAC2, fob1::LEU2/FOB1, rad52Δ::hphMX/RAD52*
HFY5	*MATa/α, cac3Δ::kanMX/CAC3, fob1::LEU2/FOB1, rad52Δ::hphMX/RAD52*
HFY18	*MATa/α, pol30Δ::kanMX/POL30, pBL230-0*
HFY19	*MATa/α, pol30Δ::kanMX/POL30, pBL230-6*
HFY20	*MATa/α, pol30Δ::kanMX/POL30, pBL230-8*
HFY21	*MATa/α, pol30Δ::kanMX/POL30, pBL230-79*
HFY32	*MATa, cac1Δ::hphMX*
HFY33	*MATa, cac2Δ::hphMX*
HFY34	*MATa, cac3Δ::hphMX*
HFY70	*MATa, E-proΔ::GAL1/10-URA3*
HFY73	*MATa, E-proΔ::GAL1/10-URA3, cac1Δ::kanMX*
HFY76	*MATa, E-proΔ::GAL1/10-URA3, sir2Δ::kanMX*
HFY106	*MATa, MCD1-6H10FLAG::kanMX, sir2Δ::URA3*
HFY122	*MATa, MCD1-6H10FLAG::kanMX, cac1Δ::hphMX*
MSY45	*MATa*
MSY360	*MATa, NatNT2-GALL-FOB1, bar1::LEU2*
MSY426	*MATa, MCD1-6H10FLAG::kanMX*
MSY613	*MATa, cdc9::tetO7-CDC9, cmv_LacI-natr, bar1::LEU2*
MSY713	*MATa, mre11-H125N*
MSY920	*MATa, sir2Δ::kanMX*
MSY937	*MATa, NatNT2-GALL-FOB1, bar1::LEU2, sir2Δ::hphMX, hmlΔ::kanMX*
MSY1231	*MATa, NatNT2-GALL-FOB1, bar1::LEU2, cac2Δ::kanMX*
MSY1261	*MATa, NatNT2-GALL-FOB1, bar1::LEU2, cac1Δ::kanMX*
MSY1262	*MATa, NatNT2-GALL-FOB1, bar1::LEU2, cac3Δ::kanMX*
MSY1376	*MATa/α, cac1Δ::hphMX/CAC1, YCp22*
MSY1377	*MATa/α, cac1Δ::hphMX/CAC1, YCp22-CAC1*
MSY1380	*MATa/α, cac1Δ::hphMX/CAC1, YCp22-cac1-20*
MSY1445	*MATa/α,, NatNT2-GALL-FOB1/FOB1, bar1::LEU2/BAR1, cac1Δ::hphMX/CAC1, mre11Δ::kanMX/MRE11*
MSY1446	*MATa/α,, NatNT2-GALL-FOB1/FOB1, bar1::LEU2/BAR1, cac1Δ::hphMX/CAC1, sae2Δ::kanMX/SAE2*
MSY1550	*MATa/α, cac1Δ::hphMX/CAC1, cac2Δ::kanMX/CAC2, cac3Δ::klTRP1/CAC3*
MSY1599	*MATa, cdc9::tetO7-CDC9, cmv_LacI-natr, bar1::LEU2, cac1Δ::hphMX*
MSY1802	*MATa/α, cac2Δ::kanMX/CAC2, mre11Δ::klTRP/MRE11*
MSY1811	*MATa/α, cac1Δ::kanMX/CAC1, exo1Δ::klTRP/EXO1, sgs1Δ::hphMX/SGS1*
MSY1886	*MATa, cac1Δ::hphMX, YCp22*
MSY1890	*MATa, cac1Δ::hphMX, YCp22-CAC1-9myc*
MSY1894	*MATa, cac1Δ::hphMX, YCp22-cac1-20-9myc*

MSY1909	*MATa/α, cac2Δ::kanMX/CAC2, sae2Δ::klTRP/SAE2*
MSY1910	*MATa, cac1Δ::kanMX, mre11-H125N*
MSY1915	*MATa, cdc9::tetO7-CDC9, cmv_LacI-natr, bar1::LEU2, cac3Δ::klTRP1*
MSY1917	*MATa, cdc9::tetO7-CDC9, cmv_LacI-natr, bar1::LEU2, cac2Δ::hphMX*

All strains are derivatives of W303, which is *ade2-1, ura3-1, his3-11, 15, trp1-1, leu2-3, 112, can1-100*, *rad5*. MSY613 was a *bar1::LEU2* derivative of yIW310, which is a kind gift of Iestyn Whitehouse. MSY713 was a kind gift of Lorraine Symington.
